# An Update on Deaths in the United Kingdom from ‘Poppers’ (Alkyl Nitrites), with a Particular Focus on ‘Swallowing’ Fatalities

**DOI:** 10.3390/jcm14020427

**Published:** 2025-01-10

**Authors:** John Martin Corkery, Caroline S. Copeland, Stephen Ream, Peter Streete, Fabrizio Schifano

**Affiliations:** 1 Psychopharmacology, Drug Misuse and Novel Psychoactive Substances Research Unit, School of Life and Medical Sciences, University of Hertfordshire, College Lane Campus, Hatfield, Hertfordshire AL10 9AB, UK; j.corkery@herts.ac.uk; 2 Institute of Pharmaceutical Science, King’s College London, London SE1 9NH, UK; caroline.copeland@kcl.ac.uk; 3 Re-Solv, Philanthropy House, Priestly Court, Staffordshire Technology Park, Stafford ST18 0LQ, UK; director@re-solv.org; 4 Hampshire Scientific Service, Hyde Park Rd, Southsea, Hampshire PO5 4LL, UK; peter.streete@btopenworld.com

**Keywords:** ‘poppers’, alkyl nitrites, deaths, swallowing, oral ingestion, United Kingdom

## Abstract

**Background/Objectives:** Alkyl nitrites are a class of inhalant, commonly known as ‘poppers’. Although having medical uses, some other effects include a ‘rush’, ‘high’, ‘euphoria’, or feeling of excitement. This has led to their recreational use, in different scenarios, since the mid-1960s. Adverse effects include tachycardia, migraine headaches, fainting and dizziness, and ventricular fibrillation. Death can occur from the inhalation or ingestion of nitrites. As part of its updated advice to the United Kingdom (UK) Government, the Advisory Council on the Misuse of Drugs considered popper-related mortality, seeking an accurate estimate of deaths. **Methods:** Data from a range of sources, including specialist mortality databases, were collated and analysed in terms of the key characteristics of decedents and fatal incidents, including the use mode. The chemical names of the nitrites were used in searches. **Results:** At least forty-two deaths occurred during 1987–2018; two were female. The mean age at death was 44 (range of 20–75) years. Most were White. Most fatalities occurred in England. The specific nitrites mentioned (N = thirty-two) were isobutyl (fourteen); amyl (seven); isopropyl (six); alkyl (three); and butyl (two). The mode of use was only known in 23/42 cases. The product was definitely swallowed in five cases, and very likely in a further one. Four additional cases were identified from the literature and media searches. **Conclusions:** The lack of a current systematic identification of relevant deaths and shortcomings in historical specialist mortality databases have severely limited what could be established with certainty about these cases. The same criticisms also apply to inhalant mortality data more generally. Nevertheless, the information presented here allows for some conclusions to be drawn and inform UK policy development.

## 1. Introduction

### 1.1. Alkyl Nitrites

Alkyl nitrites are a class of inhalant comprising the following substances: amyl nitrite (isoamyl nitrite, isopentyl nitrite); butyl nitrite (n-butyl nitrite); hexyl nitrite; isobutyl nitrite (2-methylpropyl nitrite); isopropyl nitrite (2-propyl nitrite); and pentyl nitrite (n-pentyl nitrite). These are substances which can be easily synthesised as liquids and contain a nitrite group (-O-N=O) linked to different hydrocarbon or alkyl chains; for more details, see Annex A of the recent ACMD report [1]. The nitrite group can release nitrogen oxide (NO) when within the human body; this molecule is key in smooth muscle relaxation, including in blood vessels (see below).

Alkyl nitrites commonly go by the name of ‘poppers’ as they were originally sold in glass vials (or ‘pearls’) which popped when opened to release the vapours from the liquid contents. Subsequently, these products appeared for sale as “small, colourful sealed bottles …, [though] [m]ore recently, brown screw-top bottles have been encountered, that sometimes contain a small white (probably plastic) ball which may be included as a desiccant or an agitator. ‘Poppers’ containers may or may not be tamper-proof …“ [1]. These products are highly volatile liquids, ranging from colourless to pale yellow.

Alkyl nitrites have had medicinal uses, e.g., for heart problems such as angina pectoris [2] and treating cyanide poisoning [3]. This is particularly true of amyl nitrite. Amyl nitrite should not be confused with amyl nitrate, which has very different properties, e.g., as a reagent in organic syntheses [4] and an additive in diesel fuel [5]. Amyl nitrite is also used as a solvent and cleaning agent in both domestic and industrial settings.

At room temperature, many compounds of this class of substance are sufficiently volatile to allow for their inhalation. When inhaled, alkyl nitrites cause the rapid (up to 30 s) but brief (5–10 min) non-specific relaxation of the smooth muscles surrounding blood vessels, allowing the latter to dilate, thereby lowering the blood pressure [1]. To compensate for this change, an increase in the heart rate or reflex tachycardia occurs [6]. Other effects caused by vasodilation include dizziness or giddiness; flushing; a headache; nausea; vomiting; and a ‘rush’, ‘high’, ‘euphoria’, or feeling of excitement and heat [7,8,9,10]. Accordingly, poppers have been used recreationally since the mid-1960s [11], originally largely within the homosexual community to enhance sexual encounters [12,13] before spreading to ‘avant-garde’ heterosexual groups during the 1970s [14]. In the United Kingdom (UK) context, nitrites have also been used in the context of the disco/club/rave scenes since the 1970s [7].

### 1.2. Availability of Poppers

There is little up-to-date information available on the prevalence of ‘popper’ use in the UK. The Crime Survey for England and Wales (formerly the British Crime Survey) reports information for the period from 1995 to 2015/16 only [15]. Data for the latest year(s) available indicate a lifetime use amongst 16–59-year-olds of 8.3% compared to 5.2% of 16–24-year-olds in 2015/6; the last year use rates were 0.5% and 0.7%, respectively, in 2016/7. These rates translate into estimated numbers of lifetime users of 2,730,000 aged 16–59 years and 318,000 aged 16–24 years; for last year use, the respective estimates were 169,000 and 73,000. It should be noted that prevalence rates were considerably higher around 2009. The Scottish Crime and Justice Survey [16] indicates that in 2019/20, 1.2% of respondents had used poppers in the last year (males, 1.4%; females, 1.0%; 16–24-year-olds, 5.0%). Of all those who had taken drugs in the last year, 8.9% stated they had used poppers (males, 8.4%; females, 9.4%; 16–24-year-olds, 17.2%). The latest statistics for Northern Ireland [17] indicate that amongst 15–64-year-olds, the lifetime use was 7.4% and the last year use was 1.1% in 2014/5. Lifetime rates were highest for 25–34-year-olds (14.8%), followed by 35–44-year-olds (9.3%) and 15–24-year-olds (7.0%). Last year use exhibited a different pattern: 2.7% for 15–24-year-olds, followed by 1.7% for 25–34-year-olds, and 0.6% for 35–44-year-olds. Rates tended to be higher for males than females.

Additional information is available from the English Festival Survey (EFS). Pooled data from surveys taken in 2016 and 2019 (N = 964) indicated the last year use as 8.3%. Whilst 46.5% stated they were male, 52.6% female, and 0.3% transgender, the self-ascribed sexual identity was given as heterosexual (‘straight’) by 89.4%, gay/lesbian by 4.8%, and bisexual by 4.5% [18]. EFS data for the period 2014–2023 (N = 11,566) reveal that heterosexual males were 1.56 times more likely to use poppers compared to heterosexual females, with this being 1.43 for lesbians and 1.80 for bisexual females. Homosexual males (5.81) and bisexual males (4.43) were also more likely than the comparison group to use these products [19].

Poppers have been and are sold with a range of ‘brand’ names, including ‘Bang Aroma’, ‘Brain Drain’, ‘Buzz Aroma’, ‘Gold’, ‘Heavy Duty Bolt’, ‘Jock’, ‘Jungle Juice’, ‘Kinky Monster’, ‘Liquid Gold’, ‘Liquid Incense’, ‘Locker Room’, ‘Oink Oink’, ‘Original Gold Aroma’, ‘Pure Gold’, ‘Ram’, ‘Rave’, ‘Rave Room’, ‘Reds’, ‘Rock Hard’, ‘Rush’, ‘Sissy’, ‘Snapper’, ’Spunk Power’, ‘Stag’, ‘Stud’, ‘Thrust’, ‘TNT’, and ‘XL Gold’.

They have been and are often deliberately passed off, packaged, and sold as other types of merchandise, such as room deodoriser, leather polish or cleaner, boot cleaner, nail polish remover, and videotape head cleaner. These ploys/approaches are employed to avoid or evade food and anti-drug laws [9,20]. Similar tactics were used by vendors of ‘legal highs’ in outlets known as ‘head shops’ [21,22].

The Food and Drug Administration (FDA) in the United States warns that “manufacturers are packaging and labeling these products in a way that may mislead consumers into thinking the poppers are safe or intended to be inhaled or ingested by drinking. They are often packaged in the same style of bottles as energy shots/drinks” [23].

Although the intended mode of use is undoubtedly sniffing or inhalation, any accompanying instructions are carefully worded, as was the case with ‘legal highs’, to belie any such mode of use, e.g., “not for human consumption”. The product (‘Liquid XL Gold Aroma’) involved in the death of a woman in Scotland is currently (April 2024) advertised on a website aimed at UK audiences as a ‘room aroma odouriser’, with 15 mL selling for £4.95. The website gives detailed instructions on how it should be used in this way. It does state that it contains isopropyl nitrite. There is no direct mention of its likely intended use, but it claims, “Just open it up and place it in a corner of the room and open the top and before you know it, you will be dancing in the sheets.” The same website also offers products using their chemical name, i.e., isopropyl nitrite and pentyl nitrite. Other websites offer amyl, pentyl, and propyl nitrites individually, as well as a combination of amyl and propyl nitrites.

There are a range of retail outlets where poppers can be purchased, including “sex shops, pubs, clubs, market stalls and sometimes tobacconists or clothes shops” [7]. They were also sold in ‘head shops’ until the passing of the Psychoactive Substances Act 2016 led to the closure of such outlets in the UK. Normally, they can be purchased from “about £1 to £5 a bottle” [7]. Poppers can easily be ordered online on the surface web and delivered to consumers’ home addresses [24]. They can be ordered from websites such as Amazon and Alibaba; the latter can also supply tert-butyl nitrite.

### 1.3. Effects of Poppers

Nitrites exert their vasodilatory effects through muscle relaxation in blood vessel walls. In cerebral blood vessels, this causes an increase in intracranial pressure, producing a euphoric effect. Nitrites were also reported to have aphrodisiac properties [25]. Indeed, they are reported to enhance the sexual experience by generating a rush or feeling of excitement [26] and are also able to relax the sphincter muscles of the vagina and anus [27,28], thereby facilitating sexual intercourse, i.e., ‘chemsex’. Positive effects of alkyl nitrite reported by homosexual men include disinhibition/relaxation during sex and more generally, sensations of dizziness and warmth, and a more pleasurable orgasm [12,29,30].

Reported negative side-effects include headaches and skin irritation [3,12]; tachycardia, migraine headaches, fainting, and dizziness [31]; and ventricular fibrillation [32]. Death can occur from the inhalation or ingestion of nitrites [33].

Inhalation is the conventional/usual way of abusing glues, solvents, gases and other volatile substances. The recommended method of using poppers is to inhale the vapours “directly from the bottle or from a cloth or cigarette dipped into the liquid” [7].

Non-inhalation routes of popper administration do exist, notably oral ingestion or swallowing—the focus of this study. However, odd instances of other routes have been reported, including an Austrian case of the intravenous injecting of isopropyl nitrite for recreational purposes, which resulted in methaemoglobinaemia that was treated successfully with toluidine blue [34]. If poppers are swallowed, cyanosis can occur due to the conversion of haemoglobin to methaemoglobin, which compromises oxygen transport [31]. Shortness of breath, fatigue, altered consciousness, and comas can also occur and are complications which may ultimately lead to death [35,36,37,38]. Intravenous methylene blue is the usual treatment for individuals with high levels of methaemoglobin [36,39].

Rarer side-effects that have, however, become of growing concern due to an increased use prevalence over the last decade or so include the induction of lipoid pneumonia, which may be caused by the accidental aspiration of amyl or butyl nitrites when inhaling them [40], and maculopathy, which is where the central (macula) region of the retina responsible for central vision is damaged [8,41,42,43].

### 1.4. Detection of ‘Poppers’

Headspace gas chromatography (GC) can be used to detect and quantify alkyl nitrites in blood [44]; GC/Fourier transform infrared spectroscopy (FTIR) can be used for the same purposes in products [45]. Moisture degrades alkyl nitrites to the corresponding alcohol, so nitrite–alcohol combinations may be detected.

Amyl nitrite is metabolised rapidly within the human body (hydrolytic denitration being the likely process); however, perhaps one-third of amyl nitrite which is inhaled is excreted in urine [46]. Following the modest inhalation of a product containing a mixture of four alkyl nitrites, present at unspecified concentrations, alcohols (such as isobutanol, isopropanol, and pentanol) stemming from alkyl nitrite metabolism were detectable in blood for less than 30 min after administration [47]. Consequently, measuring blood concentrations of nitrites (whether organic or inorganic) helps marginally when considering their involvement in poisoning or toxic incidents [44], but does indicate exposure. Screening for alkyl nitrites in clinical settings is rare [48]. Clinical and/or forensic toxicology testing require targeted analytical techniques; thus, alkyl nitrite screening is rarely undertaken. Furthermore, despite headspace GC being a regular method utilised in forensic toxicology, the technique—which is intended to reveal other substances of a volatile nature such as ethanol—would require modification to identify the targeted substances (‘poppers’). Consequently, the involvement of these compounds in suspected toxicity/drug-related deaths may not be recognised. In addition, their highly volatile nature, fast hydrolysis and metabolism, and short half-lives make identification difficult [48,49,50].

However, in a laboratory where a comprehensive volatile screening procedure was used, in 15 out of 418 of postmortem cases analysed between 1986 and 2012 that were positive for volatile components (excluding ethanol), the presence of the corresponding alcohol metabolites/degradation products were detected in blood, these being primarily associated with isobutyl nitrite or amyl or isoamyl nitrite exposure (PS (co-author), data from the Medical Toxicology Laboratory, St Thomas Hospital, London).

### 1.5. The Legal Status of Poppers in the UK

In the UK, alkyl nitrites have never been controlled under the Misuse of Drugs Act 1971. Under the Medicines Act 1968, it is illegal to advertise alkyl nitrites for sale for human consumption, but not to use them. The Advisory Council on the Misuse of Drugs (ACMD) report on Novel Psychoactive Substances was of the opinion that poppers appeared to fall within the scope of The Intoxicating Substances (Supply) Act 1985 [51]. The report contained no specific recommendations regarding alkyl nitrites.

The first detailed advice on alkyl nitrites was contained in a review of ‘poppers’ five years later, which provided an updated assessment of harms and looked at the “psychoactivity and mode of action of alkyl nitrites in the context of the Psychoactive Substances Act 2016” [52]. The ACMD’s view was that “alkyl nitrites (“poppers”) do not fall within the scope of the current definition of a “psychoactive substance” in the Psychoactive Substances Act 2016” and that “consequently [it did] not see a need for an exemption under the Psychoactive Substances Act 2016” [52]. The Council’s basic premise was that these substances do not directly stimulate or depress the central nervous system, i.e., they only have indirect physiological effects. This premise was used by an appellant in a Court of Appeal case in August 2018 concerning nitrous oxide (R v Rochester [2018] EWCA Crim 1936). However, the court took a different view and “concluded that the Psychoactive Substances Act 2016 does not require a “psychoactive substance” to be capable of producing its “psychoactive effect” by directly stimulating or depressing the central nervous system. Indirect effect is sufficient” [53].

In August 2020, the then Home Secretary, Priti Patel, noted that “As a consequence the lawfulness of the supply of poppers is uncertain. I am minded to remove this uncertainty by explicitly exempting poppers from the 2016 Act. I would seek the ACMD’s advice on an exemption” [54]. The ACMD report, published early in 2024, recommended that “alkyl nitrites should be exempted from the PSA 2016 by addition to Schedule 1 of the Act” [1].

### 1.6. Study Rationale and Aims

As part of the process of updating the harms assessment, the ACMD Working Group on Alkyl Nitrites sought to update the information on UK deaths. The filling in of this gap in the knowledge base was led by JC, supported by CSC and SM. Another member of the Working Group asked about oral ingestion, which led to the authors investigating if it was possible to identify and quantify any such cases, which led to a secondary focus on this under-investigated aspect.

The study reported here aimed to achieve the following:

Establish a more accurate estimate of the number of deaths in the UK involving ‘poppers’;
Identify what number/proportion were due to swallowing/oral ingestion;
Where sufficient information/detail on individual cases was available, to explore the characteristics of decedents and deaths;
Make relevant recommendations.

## 2. Materials and Methods

There are several sources of published information on ‘popper’ deaths in the UK.

The UK has three General Mortality Registers (GMRs): the Office for National Statistics (ONS), which covers England and Wales; the National Records of Scotland (NRS); and the Northern Ireland Statistics and Research Agency (NISRA).

Other possible sources of information were the Re-Solv newsletters and database; VSA Mortality datasets; the National Programme on Substance Use Mortality (NPSUM; formerly the National Programme on Substance Abuse Deaths [NPSAD]) database; NRS EU-MADNESS anonymised cases reported to the corresponding author; the Fatal Accident Inquiries (FAI) database [55]; the Regulation 28 reports to prevent future deaths database [56]; and TOXBASE^®^ [57].

The following literature databases were searched in July 2023 and ‘alerts’ created: Google Scholar, PubMed, and Scopus. In addition, regular searches of the Internet were conducted using the Google search engine. The search terms used included ‘death’ or ‘fatal*’ and the names of the specific nitrites listed below. To identify cases involving swallowing, the above terms were used in combination with ‘swallow*’ or ‘oral ingestion’.

Finally, searches of the British Library Newspaper Archive were conducted on 17 August 2023 by the corresponding author (JC) using the following search term combinations: ‘popper’ + ‘death’ + ‘swallow’; ‘nitrite’ + ‘death’ + ‘swallow’; ‘amyl nitrate’ + ‘death’; ’amyl nitrite’ + ‘death’; ‘butyl nitrite’ + ‘death’; ‘propyl nitrite’ + death’; ‘pentyl nitrite’ + ‘death’; and ‘cyclohexyl nitrite’ + death’.

The molecules searched for across all sources were alkyl nitrite; amyl nitrite; butyl nitrite; cyclohexyl nitrite; isoamyl nitrite; isobutyl nitrite; isopentyl nitrite; isopropyl nitrite; and propyl nitrite. Alternative (incorrect) spellings for ‘nitrite’, e.g., ‘nitrate’, were also scanned for relevance.

Further searches were conducted of the Regulation 28, FAI, and other databases in June 2024.

## 3. Results

### 3.1. Published Statistics

The ONS does not include nitrites (alkyl or other types) in its routinely published drug-related mortality statistics. However, it has published responses to three user requests for information on deaths involving alkyl nitrites, the latest covering registrations during the period 1993 to 2017 [58]. This indicates that a total of 28 deaths were registered, 15 of which only involved an alkyl nitrite. However, a more recent publication indicates that a total of 85 deaths where alkyl nitrites were mentioned on the death certificate were registered between 2001 and 2020 [59]. Neither of these statistical releases provide information on the cause of death or route of administration.

The NRS includes nitrites in Table SUB2 of the Additional Data published as part of its annual publication, “Drug-related Deaths in Scotland” [60]. During the period 2000–2022, only three fatalities where this class of substances was implicated in the death were registered: one from alkyl nitrite in 2011, one from amyl nitrate [sic] in 2020, and one from amyl nitrite in 2022. The 2011 case also involved alcohol [61]. There is no published information on the cause of death or route of administration.

The NISRA does not include nitrites (alkyl or other types) in its routinely published drug-related mortality statistics and has not published any user-requested information on this class of substances.

The other source of published statistics on deaths involving ‘poppers’ was the Volatile Substance Abuse (VSA) Mortality Project based at St George’s University of London. Although this project was set up in 1983 [62], data were retrospectively collected back to 1971. Annual reports were published until 2012, the final one covering deaths occurring in the period 1971–2009 [63]. A total of 23 deaths involving alkyl nitrites were recorded during this time frame. Some very limited information is given on the route of administration/use.

A collation of the available published statistics on UK popper deaths was attempted. However, this was a complex exercise due to the varying geographical and temporal coverage of the sources. For example, close scrutiny of all of the published VSA Mortality Project reports revealed some discrepancies between years, even allowing for retrospective updates. There are also discrepancies in the published ONS figures [58,59].

Table 1 summarises what could be collated in terms of published statistics and unpublished information.

The first identified death involving poppers occurred in 1987 and the latest in 2022.

According to the ONS, there were 28 deaths registered between 1993 and 2017 but 25 between 2001 and 2020; assuming that the most recently published statistics are correct and adding the figures published for 1993 to 2000 to those for the period 2001–2020, a total of 31 deaths registered was derived for 1993–2020. Using the NRS and EU-MADNESS data, three deaths occurred and were registered in Scotland in the period from the mid-1990s to 2023; only one death was registered in Northern Ireland between 1997 and 2022 (personal communication from the NISRA to the corresponding author, 7 May 2024). The number of deaths registered by the ONS appeared to peak around 2006–2009 with further outbreaks in 2016 and 2018.

A comparison between the published statistics and unpublished data from the VSA Mortality Project and NPSUM available to the researchers suggested that the latter sources identify more cases than do the GMRs. The specific type of alkyl nitrite involved in deaths is not published by the GMRs; neither is the mode/route of administration. The inclusion of such information was the very rare exception, rather than the rule, in the VSA Mortality Project reports.

### 3.2. VSA Mortality Project and NPSUM Cases

Collating information from the NPSUM database and the VSA Mortality Project datasets (the whereabouts of most original paper records for the latter are currently unknown) enabled some incomplete/partial characteristics of 42 deaths occurring between 1987 and 2018 to be assembled. These are presented in Table 2.

The overwhelming majority of decedents were male (40/42). The mean age at death was 44 (range of 20–75) years. Where the ethnicity was known, the majority (21/23) were White. Most of the deaths occurred in England, with one in Wales and one in Northern Ireland. Information on the type of nitrite involved in the death, found in a postmortem or forensic toxicology, was available for 32/42 cases. The specific nitrites mentioned, in descending order, were isobutyl (fourteen); amyl (seven); isopropyl (six); alkyl (three); and butyl (two).

Relatively little is known about death cases involving the swallowing or oral ingestion of poppers; this route of administration will now be focused on. Information as to whether the poppers were swallowed or taken orally was available in about half (23/42) of the cases; these products were definitely swallowed in 5 cases, and very likely in a further one. These six cases and four additional cases are examined next.

### 3.3. Swallowing Cases

Six cases of deaths arising from swallowing poppers were identified from the VSA Mortality Project and NPSUM datasets. A historic case report relating to England was identified through the literature searches, as well as one from recent media reports in Scotland, one from searches of the British Library Newspaper Archives, and one from a Regulation 28 report. A summary of the key characteristics of the decedents and the deaths is presented in Table 3 and Table 4. Further details are presented in Appendix A.

### 3.4. Characteristics of Decedents

The key characteristics of the 10 decedents are presented in Table 3. The deaths appear to have been evenly spread out, although two deaths occurred in both 2006 and 2015. Deaths have occurred in all parts of the UK except Wales; three occurred in Greater London.

Due to a lack of information being recorded or made available on these cases, it is only possible to present limited information about them. Seven out of ten were male; where the ethnicity was known, all were White/Caucasian; excluding an infant, the mean age was 41.6 (range of 20–60) years. Where known, the following suggestive characteristics were being single in terms of marital status; living with others; being employed (3/5); a known medical condition (5/6); and a history of substance use (2/4).

### 3.5. Characteristics of Deaths

The *locus* of the death was known in eight cases (see Table 4). The events leading to four deaths appear to have occurred in the decedent’s home or another’s private address; one in a hotel room; two in the street; and one in a pub (public house).

The use of alcohol appears to have been a contributory factor in three cases, possibly due to a lack of risk perception induced/affected by the alcohol. The infant’s death probably arose out of curiosity/inquisitiveness on her part concerning a container which her mother had negligently left lying around. One case resulted from confusing the container with a similar one used to contain methadone. Another case involved Cialis (Viagra) as well as a ‘popper’; this is suggestive of a sexual enhancement motivation for use. In only four cases were the decedents alone known to be using ‘poppers’, i.e., no other individual was using with them, or they were on their own.

Two deaths occurred in hospital and two individuals were declared dead on arrival at the hospital. This suggests that help was summoned, a fact underlined by the fact that, where known, half of the deaths were witnessed.

### 3.6. Results of Investigations

The underlying cause of death was known in nine cases: seven involved a ‘popper’ on its own; two involved other named substances (see Table 5). The proximal (immediate) cause of death was only known for three cases. Ingestion or swallowing was only specified in two cases. Intoxication was mentioned once, an overdose once, poisoning twice, and toxicity three times.

Limited pathological results were available: cardiological issues were mentioned in two out of three cases. Toxicology was available for seven cases: in two of these cases, other substances were detected. For the rest of the cases, information is presented on the ‘poppers’ detected. Overall, there was an even spread of nitrites implicated: amyl (three); isobutyl (four); and isopropyl (three). There was no instance of more than one nitrite being detected or implicated.

Coroners reached a range of verdicts/conclusions. Three cases were regarded as ‘Accidental’ and two as ‘Misadventure’, the latter being used by some coroners to denote the decedent undertaking an activity with known risks. In two cases, neglect was specifically mentioned. An ‘Open’ verdict was recorded in one case since the intentionality of the deceased individual was unclear. In terms of the manner of death, six of the ten deaths can be regarded as ‘accidental’.

## 4. Discussion

This study reports on ten cases of deaths in the UK resulting from the oral ingestion/swallowing of ‘poppers’, identified from a comprehensive range of sources (see above and Table 5). It was only possible to elicit sufficient information with regard to the route of administration from the VSA Mortality Project and NPSUM resources in 23 out of 42 cases (Table 2). Of these cases, six (26%) were deemed eligible for examination in this study. This suggests that the possible number of cases in these combined datasets might be about eleven, if this proportion was applied to all records from these two sources.

### 4.1. Availability of Published Information

The nature of this study necessitated the collation of information from a wide range of sources on as many UK ‘popper’-related fatalities as possible, so as to provide data that could be analysed as objectively as possible and from which scientific conclusions could be drawn appropriately.

The quality of the sources used and the information they provided exhibited some variety; the researchers feel justified in including less robust sources, as was argued in their studies looking at fatalities related to khat [64,65,66], gamma hydroxybutyrate [67], and kratom [68].

The present investigation, similarly to those previously mentioned, used information that probably lies towards the bottom of the evidential pyramid. Using, for example, a basic four-level approach [69] (1—generalisable studies; 2—conceptual studies; 3—descriptive studies; 4—single case study), some of the data used here come from the lower levels, 3 and 4. Similarly, data deployed in the present study fall across the centre of the continuum proposed by Sayre et al. [70]: metanalysis, systematic reviews, randomised controlled clinical trials, cohort studies, case–control studies, case series/case reports, editorials and expert opinions, in vivo studies, and in vitro research studies.

The current research project demonstrates that a pragmatic approach is needed to explore less well-understood events such as popper-related deaths. An improved database of properly collected and curated information could assist future studies in being higher up the evidential pyramid. The evidential levels for individual cases in the present study, in descending order, were adapted from Corkery et al. [68]: (a) documents relating to the incident investigation, the autopsy report, and the toxicology report; (b) documents relating to the incident investigation together with the autopsy report incorporating the main toxicological results; (c) a summary by the coroner/medical examiner/chief investigator; (d) a published case study; and (e) a media report.

The points made about previous research [68] bear repetition. All stakeholders—whether policy advisors, policymakers, interest groups, the media, or scientists—must be as precise as possible when presenting data. Inferences made from such information have to be based on evidence. This means the comprehensive screening and/or identification of cases; the thorough collection and accurate collation of data, especially toxicology levels, the known medical history, and the concomitant use of other substances; and the objective dissemination of information regarding deaths associated with popper use. The precise nature of any such associations in terms of the causality or contribution to death, including any caveats about the interpretation, should be made explicit. A failure to do so generates problems in the investigation and comprehension of the nature and characteristics of such events and the prevention of avoidable deaths in the future. Attempts were made to obtain sight of autopsy, pathology, toxicology and police investigation reports to reduce the issues outlined above

The potential for biases does arise from employing information from a range of sources. Unfortunately, these cannot be resolved retrospectively for the present investigation; they can be described and acknowledged. For example, more detailed pathology and toxicology information is available where there is full access to coroners’ records, but less so when dealing with media reports. Conversely, newspaper reports may be helpful in providing more details about events and motivations compared to coroners’ verdicts or the cause of death derived from death certification processes. See below for further details in the relevant subsections.

### 4.2. Information from General Mortality Registers (GMRs)

The limitations of which GMR statistics are published relating to deaths involving ‘poppers’ are outlined in the methodology. Additional searches in Registrar General annual reports revealed no further cases, mainly because of insufficient detail on specific substances, i.e., substances being subsumed under ‘solvents’, ‘volatile substances’, etc.

The current search facilities in the Regulation 28 database are limited. In order to efficiently look for potential cases involving nitrites, it was necessary to separately filter a number of report types, including alcohol, drug, and medication-related deaths; product-related deaths; suicide; etc. There was a further complication in that the names of substances were often redacted.

No detailed statistics on volatile substances, solvents, etc., are published by GMRs unless specifically requested; even then, there is often a fee payable. The ONS have not published anything recently on nitrites, and where they have, contradictory figures have been released (see Table 1). The NRS does list individual substances involved in drug poisoning deaths, but not combinations. However, the NRS and NISRA did respond to requests for information but were limited in what they could provide because of the need to protect anonymity. In order to obtain full details, researchers need to be part of a registered study, ideally sponsored/funded by a government department or agency, as was the VSA Mortality Project.

Even then, GMRs are limited in terms of what information they record, which is primarily based on the Medical Certificate of Cause of Death, which contains slightly more information than is given in a death certificate. In the case of England and Wales, the coroner’s verdict/conclusion may be available. Amongst the UK GMRs, only the NRS receives any toxicological information; this comprises only the names of substances considered by the pathologist to have been involved in the death and an indication if any other substance was mentioned in the postmortem toxicology—no levels are provided.

Obtaining named case-level detail from GMRs would be vital to approaching coroners and Procurators Fiscal for further details and access to case records—if still extant—for a retrospective study. However, such an exercise would still be likely to lack comprehensiveness as some cases might be simply recorded as a multiple drug overdose, etc. Some cases would remain only identifiable from media/newspaper reports and/or reports from toxicology providers.

### 4.3. Information from Special Mortality Registers (SMRs)

UK SMRs, such as the VSA Mortality Project, the NPSUM, and the Drug-Related Deaths Database in Scotland, have received or do receive information from a number of sources. This means that more detail on individual deaths is theoretically available to them, e.g., the socio-demographics of decedents, the past medical and substance use history, the events leading up to the death, the locus of the incident and death, the pathology and toxicology, the cause of death, the verdict/conclusion, and the manner of death. The benefits of such sources of information are self-evident, especially for identifying ‘at risk’ populations [71,72].

While the VSA Mortality Project was extant, it provided unequalled insights into deaths involving the substances it monitored and was able to flag up emerging issues such as the emergence of recreational nitrous oxide use, the use of helium and other gases as a means of committing suicide, etc. [63].

Due to the passage of time, organisational changes and moves, and a loss of institutional memories, the present study was hindered in terms of access to the VSA Mortality Project’s resources. Therefore, it was necessary to rely on the extant electronic administrative and statistical datasets. However, these also had limitations as not all the relevant information needed for this study was recorded in these records, e.g., there was a routine lack of information on the route of administration and the recording of toxicology, the product abused, etc. This means that there was a probable under-identification of cases. However, for some cases, NPSUM information was available to mitigate such shortcomings.

### 4.4. Information from the Wider Literature

There are only limited numbers of cases reported in the peer-reviewed and wider literature on deaths and ‘near misses’ involving the oral ingestion or swallowing of poppers. Relevant information from these cases is integrated within the following thematic observations.

In terms of non-UK fatalities, the authors are aware of only three possible historic cases, all involving Black males in the USA [35,73,74,75]. A more recent (2017) case was reported in the Australian media involving a 22-year-old male [76,77,78].

The number of near misses identified by the authors is somewhat higher, but such incidents appear to be rare. A total of ten overseas cases have been identified: one in the Czech Republic [79]; one in Denmark [80]; two in France [81]; one in Germany [82]; one in Switzerland [83]; and four in the USA [38,84,85,86].

A similar number (nine) of ‘near misses’ have been reported in the UK. The earliest one involved a two-year-old girl who got hold of an unlabelled bottle of “Liquid Gold” (amyl nitrite) in her mother’s bedroom and swallowed about 5 mL; following hospital treatment, she was discharged a day later [87]. A White female in her early twenties bought a 15 mL bottle of “poppers” and drank its contents [88]. Hagan and Burney [40] mention in passing a case involving a 52-year-old male who gave a history of the accidental aspiration of “poppers” (amyl or butyl nitrite). A 12-year-old boy and a 13-year-old friend bought what they thought was an ‘energy shot’ drink in a convenience store in December 2013, but it was actually “Liquid Gold” and contained amyl nitrite. One boy drank the contents but became seriously ill; fortunately, he recovered following hospital treatment [89,90]. Five individuals became ill, one seriously, after drinking poppers at the Parklife festival in Manchester during June 2015 [91]. The festival organisers warned people not to drink the products, which were being misused as shots [92].

### 4.5. Socio-Demographics

Seven-tenths of the cases reported here were male, compared to 85% and 72% of all deaths recorded, respectively, on the VSA Mortality Project [63] and the NPSUM databases for England in 2012-9 [93]. This is very close to the current tally (73%) for all NPSUM cases received by 1 July 2024. It is known that drug-related deaths are more predominant in men [94]. There are likely several different reasons for this, including that men are more likely to engage in risky behaviours [94], drug use in women is more stigmatised [95], and drugs (and alcohol) are more readily available to men [95].

The mean age at which decedents died was 37.6 (range of 1.6–60) years. This compares with 22.2 (range of 7–85) years for all VSA deaths (unpublished data) and 11 (40.11 ± 13.73) years for NPSUM deaths in 2012-9 [93]. The latter figures are close to those of all NPSUM cases received up to 1 July 2024—40.68 ± 13.40 years. These findings are also in line with the non-UK fatalities as well as the near misses (UK and overseas). Furthermore, as noted earlier, popper use was prevalent in the dance/music scene, which attracts a younger demographic, which may account for the younger average age of death following popper use than drugs in general.

Where the ethnicity was known, all decedents were White/Caucasian, in line with most individuals recorded on the VSA Mortality Project (96.8% (unpublished data)) and the NPSUM databases for England in 2013 (96.6%) [96].

Where the marital status was known, it was given as ‘single’ in all cases, whereas the proportion for ‘single’ in the VSA Mortality Project database was 85.6% (unpublished data). Where the employment status was known, 60.0% were employed, compared to 33.3% of all VSA Mortality Project cases and 33.0% of NPSUM cases in England in 2013 [96]. It is well recognised that people who dependently use drugs experience disproportionately high levels of unemployment [97]. Barriers to employment, such as employer discrimination, workplace drug testing, a criminal record, possessing limited formal education or employment skills, and chronic homelessness, exclude many from desired employment opportunities in the formal workforce [98]. This is, however, not reflected in the popper cases studied here, suggesting a different demographic of decedent, maybe due to the context of the use of poppers being more recreational drug use than dependent drug use.

### 4.6. Circumstances Leading to Death

A previous abuse history was known in only one nitrite swallowing case, compared to 87.6% of all VSA Mortality Project cases (unpublished data).

Four cases of nitrite use likely took place at a private address; this compares to 66.5% of all VSA Mortality Project cases (unpublished data).

Half of the nitrite swallowing deaths were witnessed, compared to 45.3% of all VSA Mortality Project cases (unpublished data).

Four in ten of the swallowing deaths occurred at home and an equal proportion either died in hospital or were pronounced dead on arrival; the corresponding proportions for all VSA Mortality Project cases (unpublished data) were 46.0% and 25.0%, respectively.

The use of alcohol was evident in a couple of the UK fatalities and could have contributed to a lack of awareness or appreciation of the risk. In terms of the overseas ‘near misses’, two occurred in nightclubs, one in conjunction with alcohol [81], while in the other case, the role, if any, of alcohol was unknown [76]; two occurred during house parties [80,83]); one in a homosexual sauna [82]; one took place in an “adult novelty store” [38]; and in another case, the product was mistaken for an energy drink [85]. A US fatality involved four-month-old twin boys having ingested “sweet spirits of nitre,” a folk remedy for “fussiness”, which had been added to their bottles when they developed upper respiratory tract infections; one twin died [73]. Another US fatality involved a male who had had a drink in a discotheque [35].

These outcomes reflect the possible reasons for use in three of the UK fatalities: possibly curiosity from a toddler as her mother had left it lying around; being mistaken for methadone in a similar container; and possibly to enhance sexual activity.

### 4.7. Pathology and Toxicology

Pathology details were unavailable for seven cases. Two of the three remaining cases included comments on cardiac issues (enlarged heart, left ventricular hypotrophy) and on the liver (engorged liver, fatty liver). Fatty metamorphosis was noted in a US fatality [35].

Methaemoglobin levels were only available for two cases: in case 1, in the blood (type unspecified), the level was 38%; in case 3, the peripheral blood methaemoglobin was 46%. These are at lower levels than those reported in overseas fatalities. The methaemoglobin levels recorded in the US twin were 43% haemoglobin A_1_ and 57% haemoglobin F at the time of his death [73]. The level of oxidised haemoglobin was clinically estimated at > 70% in another US fatality [35] and 95% in a third case based on toxicological analysis [75]. However, these may not be directly comparable.

The quality of the toxicological information available in these cases was variable: none was available for three cases; the presence of some substances was detected/mentioned in three cases; and in the remaining four cases, more solid information, including levels, was provided. These shortcomings largely arise from the fact that the data sources serve(d) different purposes and have varying degrees of completeness and accuracy, as well as access to key information and associated details.

The difficulties associated with the lack of standardisation across autopsy and toxicology investigations and the reporting thereof have been recognised and commented on for several decades [99]. These issues, as well as a lack of standardisation in toxicological screening, have started to be flagged up by researchers and relevant professional bodies internationally, as well as by the ACMD in several of its recent reports [100,101,102]. Government agencies and departments are currently looking to see how the situation can be improved in England and Wales [103].

The presence of an index substance does not necessarily mean that it was implicated in the cause of death. The researchers tried to ensure that the cause and effect were carefully established in individual cases. However, in rare cases, the cause of death remained “undetermined”. This is unfortunate for epidemiologists but is not unexpected based on the researchers’ collective experience.

The range of nitrites involved in the ten UK cases were evenly distributed: amyl (three), isobutyl (four), and isopropyl (three).

Five out of nine cases (55.5%) where the verdicts were given were recorded as a misadventure/accidental, with a range of other conclusions that were in line with all the VSA cases: ‘misadventure’ accounted for 44.3%, 31.7% were accidental, and the remaining third (34%) were split as follows—‘open’, 8.7%; suicide, 6.6%; any mention of solvent/volatile abuse, 4.8%; drug dependence/abuse of drugs, 3.0%; and natural causes, 0.3%.

### 4.8. Mortality Rates

The age-standardised mortality rate for deaths related to volatile substances in England and Wales remained fairly stable between 2001 and 2020, with the mean being 0.6 deaths per million population [62]. Similar information has not been published for Scotland or Northern Ireland. As far as the authors are aware, there are no published statistics on mortality rates for nitrous oxide or alkyl nitrites (‘poppers’). However, it is possible to offer estimates based on death data collected by the authors from the ACMD reports on nitrous oxide [104] and alkyl nitrites [1], the ONS mortality statistics, and prevalence estimates from the Crime Survey for England and Wales (formerly the British Crime Survey). Table 6 and Table 7 present the relevant information. The following calculation, mean annual number of deaths/estimated number of last year users, yields the following mean numbers of substance deaths per 100,000 users: volatile substances/”glues”, 1.231; alkyl nitrite/“amyl nitrites”, 0.003; and nitrous oxide, 0.004. Newcombe [105] estimated that both alkyl nitrites (poppers) and nitrous oxide had a “quite low” level of risk of death in 2021, i.e., 1 in 100,000 (1 in 50,001—500,000).

These rates are very low compared to other substances. For example, in England and Wales, the rate of deaths relating to drug misuse in 2022 was 5.39 per 100,000 people [15].

The ACMD is of the opinion that ‘poppers’ do not merit control under the Misuse of Drugs Act 1971 [1], in a similar way to their recommendation regarding nitrous oxide [104], as these volatile substances are used by relatively few individuals and historically have caused few deaths compared to other substances of abuse. Indeed, when exercises have been undertaken with respect to such controls in the UK context, these products have always been very low down in the ‘harm’ rankings used, whereby harms and risks (including morbidity and mortality) are assessed across several domains [108,109]. However, an exercise in Scotland ranked volatile substances fifth out of 19 substances [110].

Other forms of regulation continue to be necessary [1] so as to prevent and/or reduce the potential for products such as poppers getting into the hands of young people, i.e., those aged under 18 years of age. These can include restrictions on sales to those within this age group.

### 4.9. ‘Take Away’ Messages

Patterns of popper usage have not changed substantially in recent years; neither have there been any major changes in the regulation of these products within the UK. However, the product availability over the last couple of decades has increased because of the development of online shopping via the Internet and social media.

‘Poppers’ (0.003) have a similar rate of deaths per 100,000 users to nitrous oxide (0.004) but one that is considerably less than volatile substances as a whole. About one in ten deaths associated with the use of ‘poppers’ results from oral ingestion or swallowing the products; however, this is likely to be an underestimate.

The co-use of alcohol and/or psychoactive substances can impair an individual’s risk assessment concerning the use of ‘poppers’ [111,112]. This is evident in several of the cases reported here from the UK and overseas. The possibility of making an error of judgement is further compounded by the type of containers in which ‘poppers’ are sold and/or contained. A number of aspects are relevant here.

Several curious/inquisitive children, including toddlers, appear to have been capable of opening products sold as ‘room odourisors’, ‘leather cleaner’, etc., or other containers (e.g., containing methadone), resulting in fatalities and ‘near misses’. There is a clear need for retailers and others to use ‘child-proof’ containers [1]. In addition to standard ‘child proofing’, manufacturers should consider the risk of swallowing and whether the product design could be changed to prevent direct access to the contents.

Young teenagers and other young people have consumed ‘poppers’ thinking they were some sort of ‘energy shot’ or ‘energy drink’ [23,113,114]. There is a need for secondary schools and colleges to consider whether ‘poppers’ should be included as an element within the PSHE (personal, social, health, and economic) education curricula delivered in schools and colleges or in education programmes such as that delivered to university students by the Staying Safe Programme (https://stayingsafe.university/, accessed 31 December 2024).

Although some containers have wording that indicates that their contents should not be swallowed or are ‘not for human consumption’, there are several instances where this message seems to have gone unheeded. Clearer warnings need to be provided by manufacturers, retailers, and advertisers to would-be and potential consumers about the dangers of the oral ingestion and swallowing of ‘poppers’.

The Regulation 28 report by HM Coroner for County Durham and Darlington [115] includes detailed recommendations by West Yorkshire Analytical Services concerning the labelling and packaging of the product involved in Case 8 reported in Appendix A. The Department for Business, Innovations & Skills’ [116] response to the coroner’s report was to defer to the local Trading Standards Service.

### 4.10. The Strengths and Limitations of This Study

This study has several strengths. First, as far as the authors are aware, it is the largest study covering deaths resulting from the oral ingestion/swallowing of ‘poppers’. Second, it is the first study to provide an overview of the situation in the UK. Third, an early version of some of this material was included in the recent ACMD advice on ‘poppers’ [1]. Fourth, a comprehensive range of data sources known to and used by the researchers was employed.

Using all the available information from all currently available sources accessible to the researchers, Section 3.1 and Section 3.2 provide the most accurate estimates to date of popper-related deaths in the UK. The sub-set of 10 cases identified as probably associated with the oral ingestion or swallowing of poppers is likely to be an underestimate for the reasons explained above.

Severe methodological issues in terms of identifying relevant cases are caused by the lax, loose, or unqualified use of the word ‘ingestion’. It is often used instead of ‘administration’ or ‘use’, including inhalation/’huffing’. This leads to confusion and the inaccurate categorisation of cases as ‘ingestion’ as it does not necessarily mean ‘swallowing’. Those who document, record, and report cases, both deaths and ‘near misses’, should be more precise in their wording, especially for death certification processes. The recent ACMD report [1] underlines the importance of continuing to monitor deaths involving poppers.

The limitations of the UK GMRs and SMRs are several and various; they are detailed above. Ideally, this study should be replicated through identifying potential cases via the detailed screening of GMR databases and then following them up with relevant coroners, Procurators Fiscal, pathologists, and toxicologists; however, some cases may only be initially identified via media reports, as was the case with the VSA Mortality Project. However, this would be difficult to achieve retrospectively. For instance, many of the VSA Mortality Project casefiles are no longer available, electronic GMR databases have only existed since the mid-1990s, and many historic coroners’ records may have been destroyed. However, a prospective study for VSA products, including poppers, could be (re-)established for the UK with the participation of relevant stakeholders and adequate funding being made available.

## 5. Conclusions

This study has not only provided the most comprehensive summary of deaths in the UK associated with the use of alkyl nitrites (‘poppers’) to date but also presents the findings of the largest ever study looking at deaths associated with the oral ingestion or swallowing of poppers.

Despite the methodological challenges presented by the available information and data sources, it was possible to achieve the study’s aims by establishing a more accurate estimate of the number of deaths in the UK involving poppers and what proportion were due to oral ingestion or swallowing. Ten such cases were identified and detailed in respect to the characteristics of decedents and deaths. Where sufficient information is available on these cases, relevant action points or ‘take away’ messages have been outlined.

Going forward, it is recommended that improvements be made by those recording and collating data on deaths and ‘near misses’ to the level of detail noted, especially the circumstances leading to deaths or adverse experiences, pathology, toxicology (including levels), the route of administration, and the specific poppers implicated. In turn, this will inform educational and other preventative measures that could be targeted at specific ‘at risk’ populations, as identified in this study (once there is a more comparative interpretation of the decedent demographics). This may help to reduce these relatively infrequent but preventable deaths and ‘near misses’ even further. More generally, more information on deaths, hospitalisations, and treatments concerning volatile substances—let alone ‘poppers’—is needed [117].

## Figures and Tables

**Table 1:** Number of UK deaths involving ‘poppers’ by year, 1987–2022.

Year	Country and Source										
	ONS 2019 (England and Wales) Registrations	ONS 2022 (England and Wales) Registrations	NRS (2023) (Scotland) Registrations	EU-MADNESS and NRS (Scotland) Registrations and Occurrences	Media Reports and NISRA (Northern Ireland) Occurrences (Registered in 2004)	VSA Mortality Project (UK) Annual Reports (Deaths That Occurred)	VSA Mortality Project (UK) Annual Reports (Cumulative Total)	VSA Database	NPSUM Database	Amalgamated VSA and NPSUM	Confirmed Swallowing Cases (All Sources)
1987								1		1	
1988								2		2	
1989							2 (isobutyl nitrite)	2		2	
1990							5 (alkyl nitrites)	2		2	
1991							5 (alkyl nitrites)	1		1	
1992							5 (alkyl nitrites)	0		0	1 pre-July 1992
1993	1					1	6 (alkyl nitrites)	2		2	1
1994	0					1	6 (alkyl nitrites)	1		1	
1995	2						6 (alkyl nitrites)	1		1	
1996	0						6 (alkyl nitrites)	0		0	
1997	1						6 (alkyl nitrites)	0		0	
1998	0						6 (alkyl nitrites)	1		1	1
1999	1					3	9 (alkyl nitrites)	3	1	3	
2000	1					1	10 (alkyl nitrites)	1	1	2	
2001	0	0					10 (alkyl nitrites)	0		0	
2002	2	1			1		10 (alkyl nitrites)	2		2	1
2003	0	0					10 (alkyl nitrites)	0		0	
2004	0	0				1	11 (alkyl nitrites)	1		1	
2005	0	0					11 (alkyl nitrites)	0		0	
2006	0	1				3	14 (alkyl nitrites)	4	1	4	2
2007	0	1				2	16 (alkyl nitrites)	3	1	3	
2008	1	1				3 (inhalation)	21 (alkyl nitrites)	3	2	4	
2009	3	4				2 (inhalation)	23 (alkyl nitrites)	2	1	2	1
2010	0	0						1		1	
2011	3	2	1	1				2	1	3	
2012	3	1									
2013	2	1									
2014	1	1									1
2015	4	2							2	2	1
2016	3	4									
2017	0	0									
2018		5							2		
2019		0									
2020		1	1	1							1
2021											
2022			1	1							

**Table 2:** Characteristics of cases recorded by the VSA Mortality Project and NPSUM, 1987–2018.

Characteristic	Attribute	Number
Sex	Male	40
	Female	2
Age (years)	Mean = 44, range = 20–75	
Ethnicity	White	21
	UK	1
	Indian	1
	Not known/not available	19
Country of death	England	40
	Wales	1
	Northern Ireland	1
‘Swallowing’ status	Not swallowed	17
	Swallowed	5
	Possible	1
	Not known	19
Type of nitrite involved/found in postmortem/forensic toxicology	Alkyl nitrite (nitrate)	3
	Amyl nitrite (nitrate)	7
	Butyl nitrite (nitrate)	2
	Isobutyl nitrite	14
	Isopropyl nitrite	6
	Not known	10
*N*		42

**Table 3:** Key characteristics of UK decedents in deaths involving swallowing/oral ingestion of poppers.

Characteristic	Attribute	Number
Sex/gender	Male Female	7 3
Age (years)	Mean = 37.6, range of 1.6–60; excluding infant, mean = 41.6, range of 20–60	
Ethnicity	White Caucasian Not known	5 1 4
Living arrangements	With partner With flatmate Own room in shared student accommodation With mother and sibling Not known	2 1 1 1 5
Marital status	Single Not known	4 6
Employment status	Employed Unemployed Student Not known	3 1 1 5
Previous medical status	Background of heart disease Long-standing mental health issues Drug dependence None Not known	2 2 1 1 4
History of ‘popper’ use	Yes Not known	1 9
History of substance abuse, misuse, etc.	Drug and alcohol abuse Drug abuse None Not known	1 1 2 6
Year of death	<1992 1993 1998 2002 2006 2009 2015 2020	1 1 1 1 2 1 2 1
Region and country of death	Greater London, England Bedfordshire, England Buckinghamshire, England Co. Durham and Darlington, England Devonshire, England Sunderland, England Angus, Scotland Belfast, Northern Ireland	3 1 1 1 1 1 1 1

**Table 4:** Key characteristics of UK deaths involving swallowing/oral ingestion of poppers.

Characteristic	Attribute	Number
*Locus* of incident and events leading to death	Found collapsed in street; possible robbery victim.	1
	Family home.	1
	Found in room by fellow students.	1
	Possibly at home; mistook amyl nitrate (“poppers”) for methadone in a similar bottle.	1
	Was intoxicated but continued drinking; appeared to drink liquid from small bottle; found slumped in hallway	1
	Had been drinking beer and spirits in a wine bar; picked up bottle of isobutyl nitrate on table and drank it; collapsed and taken outside by friends and left there.	1
	Drinking with friends in a pub on Christmas Eve, when drank a bottle of “poppers” (isopropyl nitrite) which had purchased in local shop; collapsed and paramedics attempted resuscitation at scene.	1
	Found cold in hotel room with a small black plastic bottle under his chin; multiple areas of blood in the face, arms and torso with evacuated bowels and bleeding from the anus; empty bottle of ’amyl nitrate’ in a bin, and two pots of Viagra were found; bottle found in decedent’s mouth confirmed to be isopropyl nitrite, probably swallowed.	1
	Male customer bought 2 bottles of XL Gold from an off-licence, took them home and gave one to a female, who drunk the whole bottle; she subsequently fell ill and died same day.	1
	Not known	1
Place of death	Home address	4
	Hospital	2
	Dead on arrival at hospital	2
	Private address	1
	Hotel room	1
Death witnessed	Yes	4
	No	4
	Not known	2
Using alone	Yes	4
	Not known	6
Reason(s) for use	Possibly curiosity as mother had left it lying around	1
	Mistaken for methadone in similar container	1
	Possibly to enhance sexual activity	1
	Not known	7

**Table 5:** Results of investigations into UK deaths involving swallowing/oral ingestion of poppers.

Characteristic	Attribute	Number
Proximal cause of death	Choking and collapsing	1
	Bronchopneumonia	1
	Myocardial injury	1
	Not known	7
Underlying cause of death	Amyl nitrate toxicity	1
	Ingestion of amyl nitrite	1
	Swallowing amyl nitrite	1
	Isobutyl poisoning	1
	Isobutyl nitrite intoxication	1
	Isobutyl poisoning	1
	Drug overdose (isopropyl nitrate & illicit heroin)	1
	Likely isopropyl nitrite toxicity	1
	Combined alkyl nitrate & tadalafil toxicity	1
	Not known	1
Postmortem pathology	Bruising to face, upper limbs and back of trunk	1
	Air passages contained terminally inhaled gastric material; lungs had extensive intrapulmonary haemorrhage; enlarge heart; engorged liver	1
	Left ventricular hypotrophy and fatty liver	1
	Not known	7
Postmortem toxicology	Case 1—Bl—alcohol, 124 mg%; urine in blood, 164%; methaemoglobin in blood, 38%; 2 mg nitrite and 77 mg nitrate in stomach.	1
	Case 3—Bl—isobutyl, 1 mg/L; isobutyl nitrite and isobutyl alcohol detected; peripheral blood methaemoglobin, 46%.	1
	Case 5—Bl—256 mg/mL isobutyl detected.	1
	Case 6—Bl—2-methyl-1-propanol and isobutane detected.	1
	Case 7—Bl—methadone, 135 ug/L; benzoylecgonine, 1435 ug/L; heroin/morphine, 1374 ug/L free and 1384 ug/L total; codeine 100 ug/L.	1
	Case 9—Bl—codeine, 0.13 mg/L; diazepam + DMD + oxazepam + temazepam + diphenhydramine + paracetamol, 45 mg/L; tadalafil (Cialis), 0.18 mg/L; alcohol, 126 mg/dL; isopropanol, <10 mg/dL. Ur—codeine + diphenhydramine + alcohol, 169 mg/dL; isopropanol, <10 mg/dL.	1
	Case 10—Amyl nitrate, alcohol.	1
	Not known	3
Type of nitrite used	Amyl	3
	Isobutyl	4
	Isopropyl	3
Verdict/Conclusion	Accidental	3
	Misadventure	2
	Narrative—“died after ingesting an illegal substance—iso-butyl nitrate …”	1
	Non-dependent volatile substance abuse contributed to by neglect	1
	Adjourned; mother found guilty of neglect	1
	Open (undetermined intent)	1
	Not known	1
Intentionality/Manner of death	Accidental	6
	Dependence on volatile solvents	1
	Neglect	1
	Undetermined	1
	Not known	1
Data source(s)	NPSUM	2
	VSA	1
	NPSUM & VSA	1
	VSA & Coroner’s records	1
	NRS & newspaper reports	1
	NISRA & newspaper report	1
	Regulation 28 report	1
	Newspaper reports	1
	Published case-report	1

**Table 6:** Estimated number of individuals using volatile substances and gases, England and Wales.

Substance	Source	Period	Mean Estimated Number (000s) Using in Last Year
“Glues”	Home Office [106]	2001–2010	40.67
“Amyl nitrite”	Home Office [106]	2001–2017	328.06
Nitrous oxide	ONS [15]	2012–2014, 2016–2020, 2022–2023	704.70

**Table 7:** Number of deaths associated with volatile substances and gases, England and Wales.

Substance	Source	Period	Mean Annual Number of Deaths
Volatile substances	Ghodse et al. [63]	1971–2009	50.10
Alkyl nitrites	ONS [58]	1993–2017	1.12
Alkyl nitrites	ONS [59]	2001–2020	1.25
Alkyl nitrites	VSA Mortality Project and NPSAD (unpublished data)	1987–2022	1.11
Nitrous oxide	ONS [107]	2001–2021	2.82

## Data Availability

The data presented in this study are not available as they were obtained from coroners and other data suppliers for specific research projects and cannot be shared.

## Appendix A. ‘Swallowing’ Case Reports in Chronological Order

*Case 1.*

“A 60-year-old Caucasian male was found collapsed in a street and certified dead on arrival at hospital. The findings at the routine coroner’s autopsy indicated that death was due to natural causes. Subsequently, a second autopsy had to be carried out as it became known that his bank cash card had been used on several occasions following his death, as his death may have been linked to a criminal intent. Post-mortem examination on both occasions showed that he had ischaemic myocardial fibrous. At the initial autopsy it was noted that he had two small areas of bruising to the face, and the stomach contents had a pungent smell. At the second examination it was noted that there were multiple deep bruises to both upper limbs and the trunk posteriorily. Initial toxicological analysis showed that the deceased had consumed alcohol (blood alcohol—124 mg%, with a urine alcohol of 164%) and methhemoglobin was detected in the blood. Subsequent analysis of these samples for the purpose of instituting criminal proceedings confirmed the alcohol levels but also estimated that there was 38 per cent methhaemoglobin in the blood in addition to 2 mg nitrite and 77 mg nitrate in the stomach. The cause of death was attributed to the ingestion of amylnitrite” [118].

There is no clear indication as to whether “ingestion” in this specific case means swallowing/oral ingestion or inhalation. However, the authors do state that “Since Methaemoglobin is formed in the body, whether amylnitrite [sic] is ingested or inhaled, it would be necessary to determine the level of methaemoglobin in all cases where death is attributed to amylnitrite [sic].” This distinction could be taken to imply that in this case the amyl nitrite was taken orally.

This death occurred within the jurisdiction of HM Coroner for Greater London (Western Division)—now the equivalent of west London—some time prior to being published in July 1992. The case cannot be identified in the VSA Mortality Project database or local newspapers. Unsuccessful attempts were made to obtain further information from the authors of the case report. Coroner’s records for this period are no longer held in the coroner’s archives (personal communication to the corresponding author from the Executive Support Officer, West London Coroner’s Court, 24 June 2024).

Source: [118].
*Case 2.*

Local newspapers in Newcastle-upon-Tyne reported the death of a young female toddler, whose age was given as both 19 and 21 months. The child lived near Houghton-le-Spring with her 34-year-old mother and 6-year-old sister; her father visited occasionally. Police were called to the family home at 7.30 pm on Sunday 12 September 1993, and the toddler was taken to Sunderland General Hospital where she was pronounced dead on arrival/died shortly after arrival (depending on which report one reads). Other reports say that despite desperate lifesaving attempts by paramedics and doctors, the toddler died shortly after arrival at the hospital.

She was thought to have swallowed amyl nitrate before choking and collapsing. An initial postmortem was inconclusive. An inquest was opened but adjourned by the Sunderland coroner pending forensic tests of items taken from the family home.

The mother appeared before Newcastle Crown Court on 31 May 1995, where she denied charges of wilful abandonment of the toddler and the neglect of her 6-year-old sister. The woman had gone on a night out at a workingmen’s club and left her children in the care of two 13-year-old girls from a nearby house. The arrangement had been that the older girls would check on the youngsters from time to time, but they were otherwise unsupervised. This was not the first time this arrangement had been used. The mother claimed that she left the children with a trusted neighbour who had volunteered to babysit while she went to a local club. However, the prosecution case was that the mother was content for the neighbour’s teenage daughter to put the girls to bed, leave them alone in their own locked home, and only check on them occasionally during the night.

The 6-year-old carried her dying sister across the road to raise the alarm shortly after the pair had been bathed and put to bed by the older girls. The toddler’s skin was grey, and her body hung like a rag doll. The Crown Court was told that the postmortem showed her death had been caused by swallowing amyl nitrite [sic]. A bottle of it had been left lying around in the kitchen.

The mother was found guilty of two charges of neglect by a Crown Court jury and gaoled for 15 months to deter selfish neglect on 20 June 1995. The judge said she did not deliberately abuse her children but “ignored an obvious risk” by leaving the poppers within their reach and failing to make sure they were being properly cared for. The defence lawyer said of the mother, “she faces a regrettably barren future”.

Sources: [119,120,121,122,123,124,125,126,127], accessed via the British Library Newspaper Archive.
*Case 3.*

A White, 21-year-old European student studying at a London university had suffered from long-term depression and received help from the university’s GP surgery, being prescribed Seroxat, and had also attended a private clinic following a suicide attempt. He also received support from the student counselling service. The decedent was last seen by fellow students in the house they shared two or three days before being found dead on his bed. He was only dressed in a T-shirt and appeared to have vomited. Life was pronounced extinct at the scene. Near the body were empty cider bottles and some packets of Seroxat. A letter dated earlier that month (March 1998) from the university stated that the decedent was being excluded because of the non-payment of fees.

Pathological findings of note included air passages contained terminally inhaled gastric contents; both lungs showing an extensive intrapulmonary haemorrhage; a heart enlarged to 435 g; and an engorged liver. The toxicology showed a peripheral blood haemoglobin level of 46%; isobutyl alcohol of 1 mg/L bl; and isobutyl nitrite and isobutyl alcohol detected in non-biological samples. The pathologist stated the cause of death to be ”isobutyl nitrite poisoning”. The cause of death recorded by the coroner was “isobutyl poisoning”. Recording an “open” verdict, the coroner recorded that the “Deceased took overdose of amyl nitrate at home …”

Sources: Reported by the Poisons Unit in February 2001 to the VSA Mortality Project; a copy of the case papers was provided by HM Coroner for inner south London (unpublished data).
*Case 4.*

A male, aged 25, was found in an intoxicated state by his flatmate at about 5.25 pm in March 2002. The decedent was described as looking ashen and grey but carried on drinking. He appeared to drink liquid from a small bottle. At about 6 pm, the decedent said he was going to bed, but a short time later his flatmate found him slumped in the hallway and called an ambulance, but he was dead before it arrived. The Belfast inquest heard that the decedent had a history of drug and alcohol abuse, as well as a long history of mental health issues, including paranoia. He had appeared to be improving in the months leading up to his death, although he missed mental health appointments and his compliance with medication was erratic. Cause of death: 1a, isobutyl nitrite intoxication. The coroner concluded that the decedent “died after ingesting an illegal substance, iso-butyl nitrate, a drug not reckoned to be a particularly serious one, she said, but ingestion had caused death in the past“ [110]. The death was registered as the International Classification of Diseases code F18.2 (“Mental and behavioural disorders due to use of volatile solvents: dependence syndrome”).

Sources: [128]; Northern Ireland Statistics and Research Agency (unpublished data).
*Case 5.*

A white male, born May 1953, died November 2006, aged 53. He was employed and lived with a partner. He had been drinking Tetley Bitter and Bacardi and Coke in a pub/wine bar and was extremely drunk. Whilst in the pub, there was a bottle of isobutyl nitrate on the table, which he then drank in one go. A short while later, he collapsed, was taken outside, and was left unconscious for almost one and a half hours before an ambulance was called. All attempts to revive him failed and he died in hospital. He was not on prescribed medications, with no drug abuse history. Cause of death: 1a, bronchopneumonia; 1b, cerebral anoxia; 1c, isobutyl nitrite [sic] poisoning; 2, acute alcoholic intoxication. Toxicology: alcohol, 256 mg/mL; isobutyl nitrite was detected. An inquest was held in southwest England; the verdict was “Accidental” and non-dependent volatile substance abuse contributed to by neglect.

Sources: a Re-Solv newspaper clipping, the VSA Mortality Project, and the National Programme on Substance Abuse Deaths (unpublished data).
*Case 6.*

White male, born 1963, died in December 2006, aged 43. Employed in a skilled trade, single. Died at home. Was using alone. Previous use history unknown. Product not specified but administered directly into the mouth. Toxicology: 2-methyl-1-propanol, isobutane. Inquest held in east of England; verdict was “Accidental”.

Source: VSA Mortality Project (unpublished data)
*Case 7.*

White female, born October 1977, died February 2009, aged 31. Unemployed, lived with partner. Died in hospital. Cause of death: 1a, myocardial injury; 1b, drug overdose (isopropyl nitrate and illicit heroin). Toxicology: methadone, bl, 135 ug/L; benzoylecgonine, 1435 ug/L; heroin/morphine, bl, 1374 ug/L free and 1384 ug/L tot; and codeine, bl, 100 ug/L. Mistook amyl nitrate (“poppers”) for prescribed methadone in similar bottle. Ambulance crew called but failed to respond. Deceased and partner regular users of heroin, cocaine, and crack. On prescribed methadone. Inquest held in east of England; verdict: “Accidental”.

Source: National Programme on Substance Abuse Deaths (unpublished data).
*Case 8.*

“A 49 year old man (with a background of heart disease: Left Ventricular Hypertrophy, Coronary and Valvular) was drinking in the pub on Christmas Eve (24.12.14) with friends when he drank a bottle of “Poppers” (isopropyl nitrite) which he had purchased in a local shop. He collapsed and Paramedics attempted resuscitation at the scene. He was taken to … Hospital where he was pronounced dead. He had previously ingested “Poppers” without ill effects.” … “The conclusion of the inquest [completed on 13 April 2015 by the northeast England coroner] was Misadventure. The medical cause of death was: 1a) Likely Isopropyl Nitrite toxicity[;] 2) Left Ventricular Hypertrophy and Fatty Liver”.

“Isopropyl nitrite is sold as a liquid in a small (25 mL) bottle as “English Room Odoriser”. It is known that some inhale the vapour which is thought to cause a short-lived “rush”/euphoria. The label on the bottle says” do not inhale”. [The coroner referred to a] report from West Yorkshire Analytical Services which states “The labelling was examined with respect to the Chemicals (Hazard Information and Packaging for Supply) Regulations 2009 (CHIP) and the Regulation Classification, Labelling and Packaging of Substances and Mixtures 2008 (CLP) with the following observations:

Need upgrading for CLP to include the signal word “danger”. Pictograms need updating to CLP standard. Need to include the hazard statements suggested H225 Highly Flammable Liquid and Vapour, H301 Toxic if Swallowed, H331 Toxic if Inhaled. Precautionary statements suggested P210 Keep Away From Heat/Sparks/Open Flames/Hot Surfaces—No Smoking, P261 Avoid Breathing Vapours, P301 and P310 IF SWALLOWED: Immediately call a POISON CENTRE or Doctor/Physician. The container requires a tactile warning”.

A copy of the coroner’s Regulation 28 report to prevent future deaths was sent to the Department of Business, Innovations and Skills, General Product Safety Department, 1 Victoria Street, London, SW1H 0ET. The response on 11 May 2015 from the European Reform Directorate was to refer the coroner to the local Trading Standards Authority.

Source: [115].
*Case 9.*

A male, aged 42, of unknown ethnicity, and employed (non-manual), died in a southwest London hotel in October 2015. He was found cold with a small black plastic bottle under his chin. There were multiple areas of blood in the face, arms, and torso with evacuated bowels and bleeding from the anus. There was an empty bottle of ‘amyl nitrate’ in a bin and two pots of Viagra. A bottle found in the decedent’s mouth was confirmed to be isopropyl nitrite by the homicide assessment team. This substance explained the stool and bleeding from the anus. It is probable that he swallowed the isopropyl nitrite. The decedent’s drug use status was unknown; he was not known to be on prescribed medication. Cause of death: 1a, combined alkyl nitrate and tadalafil toxicity. Toxicology: codeine, 0.13 mg/L bl and ur; diazepam, bl; DMD (dimethyldiazepam), bl; oxazepam, bl; temazepam, bl; diphenhydramine, bl and ur; paracetamol, 45 mg/L bl; tadalafil (Cialis), 0.18 mg/L bl; alcohol, 126 mg/dL bl, 169 mg/dL ur; isopropanol, <10 mg/dL bl, <10 mg/dL ur. A verdict of misadventure was recorded by the west London coroner; the manner of death was regarded as accidental.

Source: National Programme on Substance Abuse Deaths (unpublished data).
*Case 10.*

A male purchased two bottles of XL Gold from an off-licence (corner shop) in Angus, Scotland. He took them home and gave one to his girlfriend—the decedent—who was White and 51 years old. She drank its entire contents, became ill, and died the same day in May 2020. XL Gold usually contains isopropyl nitrite. The cause of death registered by the National Records of Scotland (NRS) was “Amyl nitrate toxicity”. The postmortem toxicology also indicated the presence of alcohol. The death was recorded as the International Classification of Diseases 10 code X44—accidental poisoning by and exposure to other and unspecified drugs, medicaments, and biological substances.

A licencing officer visited the shop in July 2020 and interviewed the licence holder. It appears that the licensee had stocked the product for at least 12 months and “understood that the point of the product was for the smell”. When the licencing officer visited, bottles of XL Gold were “displayed on the counter among miniature bottles of alcohol but [the licensee] said it was normally kept behind the counter”. The licencing officer’s report added, “I asked if it is possible that this product has ever been sold as or advertised as alcohol. He said that it is not the case, and it has always been clear that it is not alcohol. He told me, however, he does accept that the box being on the counter instead of being packed away as it normally was, may have caused confusion on the day in question. He also told me that the bottle has clear indications on it that it should not be consumed, with prominent skull and crossbones on the bottle, as well as a clear danger message and the actions that should be carried out if the product is consumed.” (Jeffay and King, 2020).

Sources: [129]; National Records of Scotland (unpublished data).
